# MicroRNAs and Neutrophil Activation Markers Predict Venous Thrombosis in Pancreatic Ductal Adenocarcinoma and Distal Extrahepatic Cholangiocarcinoma

**DOI:** 10.3390/ijms21030840

**Published:** 2020-01-28

**Authors:** Julia Oto, Silvia Navarro, Anders C. Larsen, María José Solmoirago, Emma Plana, David Hervás, Álvaro Fernández-Pardo, Francisco España, Søren R. Kristensen, Ole Thorlacius-Ussing, Pilar Medina

**Affiliations:** 1 Haemostasis, Thrombosis, Atherosclerosis and Vascular Biology Research Group, Medical Research Institute Hospital La Fe (IIS La Fe), 46026 Valencia, Spain; juliaotomartinez@gmail.com (J.O.); navarro_silros@gva.es (S.N.); sol_mjo@gva.es (M.J.S.); plana_emm@gva.es (E.P.); alvarofernandezpardo@gmail.com (Á.F.-P.); espanya_fra@gva.es (F.E.); 2 Department of Gastrointestinal Surgery and Centre of Clinical Cancer Research, Aalborg University Hospital, 9000 Aalborg, Denmark; anchl@rn.dk (A.C.L.); otu@rn.dk (O.T.-U.); 3 Angiology and Vascular Surgery Service, La Fe University and Polytechnic Hospital, 46026 Valencia, Spain; 4 Data Science, Biostatistics and Bioinformatics Unit, Medical Research Institute Hospital La Fe (IIS La Fe), 46026 Valencia, Spain; bioestadistica@iislafe.es; 5 Department of Clinical Biochemistry, Aalborg University Hospital, 9000 Aalborg, Denmark; srk@rn.dk; 6 Department of Clinical Medicine, Aalborg University, 9000 Aalborg, Denmark

**Keywords:** pancreatic ductal adenocarcinoma, distal extrahepatic cholangiocarcinoma, microRNA, neutrophil, venous thromboembolism, calprotectin, biomarker, thrombosis, NETosis

## Abstract

Cancer-associated venous thrombosis (VTE) increases mortality and morbidity. However, limited tools are available to identify high risk patients. Upon activation, neutrophils release their content through different mechanisms, thereby prompting thrombosis. We explored plasma microRNAs (miRNAs) and neutrophil activation markers to predict VTE in pancreatic ductal adenocarcinoma (PDAC) and distal extrahepatic cholangiocarcinoma (DECC). Twenty-six PDAC and 6 DECC patients recruited at cancer diagnosis, were examined for deep vein thrombosis and pulmonary embolisms, and were then followed-up with clinical examinations, blood collections, and biCUS. Ten patients developed VTE and were compared with 22 age- and sex-matched controls. miRNA expression levels were measured at diagnosis and right before VTE, and neutrophil activation markers (cell-free DNA, nucleosomes, calprotectin, and myeloperoxidase) were measured in every sample obtained during follow-up. We obtained a profile of 7 miRNAs able to estimate the risk of future VTE at diagnosis (AUC = 0.95; 95% Confidence Interval (CI) (0.987, 1)) with targets involved in the *pancreatic cancer* and *complement and coagulation cascades* pathways. Seven miRNAs were up- or down-regulated before VTE compared with diagnosis. We obtained a predictive model of VTE with calprotectin as predictor (AUC = 0.77; 95% CI (0.57, 0.95)). This is the first study that addresses the ability of plasma miRNAs and neutrophil activation markers to predict VTE in PDAC and DECC.

## 1. Introduction

Venous thromboembolism (VTE) is a condition in which blood clots form most often in the deep veins of the leg, known as deep vein thrombosis (DVT). When this blood clot is disrupted from the vessel wall, it can travel in the circulation and lodge in the lungs, thereby causing a pulmonary embolism (PE). VTE is a common complication of cancer patients often leading to a reduced survival [1], and treatment of cancer patients with VTE results in significantly increased treatment costs and reduced quality of life [2].

Epidemiologic studies have confirmed that pancreatic cancer patients have a high incidence of VTE [3], with 4.3 VTE events per 100 hospitalizations [4]. In pancreatic cancer, VTE is associated with a median overall survival of 5.8 months in patients with VTE vs. 10.3 months in patients without VTE (*p* = 0.031). The overall survival is even worse when the VTE event occurs during chemotherapy [5]. Furthermore, metastatic pancreatic cancer patients have a 2.1-fold higher risk for recurrent VTE than other metastatic cancer patients.

Distal extrahepatic cholangiocarcinoma (DECC) is anatomically closely related to pancreatic ductal adenocarcinoma (PDAC) [6]. DECC can often not be separated from PDAC unless the patients undergo radical surgery and even then the differentiation can be difficult [7,8]. Furthermore, the risk of VTE in patients with cholangiocarcinoma is almost as high as in pancreatic cancer patients with a comparable low survival [9].

Several clinical assessment scores have been proposed for thrombotic risk stratification in cancer patients [10,11,12,13,14,15]. The Khorana score is commonly used in predicting the risk of VTE in chemotherapy treatment [10]. As for all clinical assessment scores there are limitations [12,16,17,18,19,20] and current tools for predicting and monitoring the risk of VTE are inadequate, especially in pancreatic cancer [21]. The discovery of novel and precise biomarkers to identify cancer patients with a high risk of VTE could either, replace or strengthen a clinical risk score.

microRNAs (miRNAs) are small non-coding RNAs that regulate protein expression. They are identified as regulatory molecules and biomarkers in virtually all cancer types, and in pancreatic cancer, several miRNAs could turn out to be valuable biomarkers [22,23,24,25,26]. Despite an extensive bibliographic revision, there is, to our knowledge, no literature on miRNA and risk of VTE in PDAC or DECC patients.

Neutrophil granulocytes are the most abundant type of white blood cells in the immune system. Upon activation, they play a prominent role in defense mechanisms by phagocytosis, degranulation and by neutrophil extracellular trap (NET) formation. NETs are extracellular networks of DNA, histones and granule proteins (calprotectin, myeloperoxidase, elastase, etc.) released by neutrophils in response to an inflammatory stimulus [27] or to the presence of pathogens, in a process called NETosis [28]. NETs may trigger coagulation and, in turn, increase the risk of VTE. In cancer-associated thrombosis, cancer cells activate neutrophils to produce more NETs than those activated by other means [29,30]. Pancreatic cancer cells can stimulate the rapid release of NETs, which promote thrombus formation under venous shear stress ex vivo [31]. Boone et al. demonstrated in a murine model of pancreatic cancer that NETs promote hypercoagulability, which is diminished by chloroquine [32].

In a prospective study, which included patients with a suspected upper gastrointestinal cancer, the patients with PDAC and DECC were examined at time of cancer diagnosis and followed for two years with blood samples and VTE examination every third month. We aimed to identify a profile of plasma miRNAs and markers of neutrophil activation, in order to predict a VTE event in PDAC and DECC. In addition, plasma was investigated for up- or down-regulated miRNAs in the last blood sample before the VTE event, and then compared with the analysis of the blood sample at inclusion in an attempt to identify one or more mechanisms triggering VTE in PDAC and DECC.

## 2. Results

### 2.1. Clinical Characteristics of the Study Subjects

Among the 121 cancer patients recruited in the original study [33], a VTE event was objectively diagnosed in 15 patients (12.4%) at the time of cancer diagnosis and, due to the study protocol of the present study, excluded. During follow-up 10 patients (8.3%) developed a VTE event. The characteristics of patients and age- and sex-matched controls are listed in Table 1, and those of the original cohort of 121 cancer patients can be found in the original study [33]. Three patients developed DVT and two developed pulmonary embolism. Five patients developed DVT and PE simultaneously during follow-up.

### 2.2. miRNA Expression Levels: Screening Stage

Based on the quality of the isolated RNA, we selected five patients who suffered a VTE event during follow-up (VTE group) and 5 who did not. These selected patients were matched on age and sex. In them, the expression level of 179 miRNAs commonly found in human plasma was studied, at inclusion and also right before the VTE event for the VTE patients. We obtained high quality signals (Ct < 36) in 123 of the 179 miRNAs included in the panel in at least one study group. Regarding the synthetic RNA controls for RNA isolation, cDNA synthesis and inter-plate performance we did not observe differences between both clinical groups (data not shown).

With respect to the best endogenous reference to normalize the expression level of each miRNA, the comprehensive tool RefFinder rendered miR-93-5p as the one with the highest stability and the lowest biological variance among all samples. As a result, we normalized every miRNA expression level using the miR-93-5p as endogenous reference by the 2-ΔΔCT method.

Our main goal was to identify a miRNA profile at diagnosis able to predict the occurrence of a VTE event in PDAC and DECC cancer patients during follow-up. To that end, in this screening stage, we adjusted an elastic net logistic regression model for VTE risk using the miRNA expression levels at inclusion. It comprised the expression level of 11 miRNAs as predictors: miR-486-5p, miR-32-5p, miR-106b-5p, miR-326, let-7i-5p, let-7g-5p, miR-144-5p, miR-144-3p, miR-19a-3p, miR-103a-3p and miR-30e-3p (Table 2). These miRNAs had a fold-change ranging from −2.58 to 4.28.

Furthermore, we aimed to identify up- or down-regulated miRNAs in the sample right before the VTE event compared with that obtained at inclusion, since this could shed light on the mechanism triggering a VTE event in PDAC and DECC patients. We identified a profile of 7 down-regulated miRNAs (miR-30e-3p, let-7i-5p, let-7g-5p, miR-144-3p, miR-199a-3p, miR-101-3p and miR-15a-5p) that might prompt the VTE event in these patients during follow-up (Table 3). Provided that this statistical analysis corresponds to paired samples, a delta ought to be calculated instead of a fold-change. Delta represents the difference in the expression level of a given miRNA between the sample at inclusion and the one right before the VTE event. The greatest difference in the delta values were found in miR-144-3p (delta: −0.8) and let-7g-5p (delta: −0.34). It could indicate a greater involvement of these two miRNAs in triggering the VTE event in PDAC and DECC patients.

### 2.3. miRNA Expression Levels: Confirmation Stage

**Figure 1:**
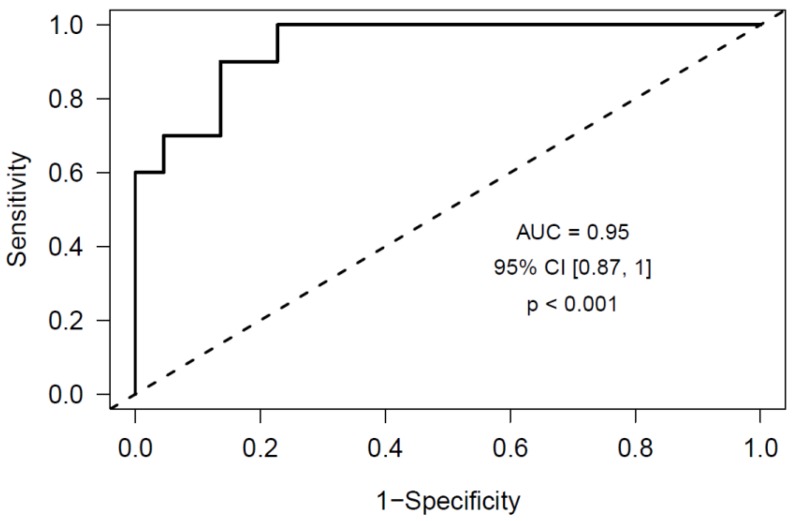
Receiver operator characteristic ROC curve obtained from the confirmation data set using the Elastic Net model that includes 7 miRNAs (miR-486-5p, miR-106b-5p, let-7i-5p, let-7g-5p, miR-144-3p, miR-19a-3p and miR-103a-3p) as risk predictors of future cancer-associated venous thrombosis (VTE) in pancreatic ductal adenocarcinoma (PDAC) and distal extrahepatic cholangiocarcinoma (DECC) patients at diagnosis.

Next, we aimed to confirm the predictive ability of our model in a larger cohort of PDAC and DECC patients at inclusion, 10 who developed a VTE during follow-up and 22 who did not. In these, we could quantify by RT-qPCR the expression level of 7 of the 11 miRNAs comprised in the predictive model: miR-486-5p, miR-106b-5p, let-7i-5p, let-7g-5p, miR-144-3p, miR-19a-3p and miR-103a-3p. The remaining 4 miRNAs had a very low expression level and did not achieve a suitable qPCR criteria (Ct < 35 and SD between duplicates <0.5). In this confirmation stage, we obtained a receiver operator characteristic ROC curve AUC of 0.95 in this cohort of 32 patients (95% Confidence Interval (CI) (0.87, 1), *p* < 0.001) (Figure 1). The formula for estimating the thrombotic risk in PDAC and DECC patients with our model is as follows (Formula (1)):

(1) $$\mathrm{Pr}\left(Thrombosis\right)=\frac{e^{LP\;\;}}{1+e^{LP\;}}$$

where LP is:

$$\begin{array}{c} LP=-0.65\;+\;0.28*miR486\_5p\;+\;1.97*miR106b\_5p\;-1.12*miR19a\_3p-\\ 5.23*miR103a\_3p\;-9.67*let7i\_5p\;+\;1.8*let7g\_5p\;+8.61*mir144\_3p \end{array}$$

Furthermore, we estimated the thrombotic risk of each patient at inclusion by applying the aforementioned formula of our predictive model. The median thrombotic risk at inclusion of the group of patients who suffered a VTE during follow-up was 0.72, while it was only 0.13 in the group of patients who did not suffer a VTE during follow-up (*p* < 0.0001).

### 2.4. Identification of the miRNAs’ Targets

We identified a profile of 7 miRNAs able to accurately predict at inclusion a VTE event in PDAC and DECC patients during follow-up. Next, we identified their validated and predicted target proteins related to cancer and VTE by using the database miRWalk 2.0. This database integrates computational algorithms for target identification rendering two types of targets: Predicted targets were those being theoretically estimated based on the free binding energy between a miRNA and a putative target mRNA sequence; and validated targets were those empirically validated to be regulated by a miRNA. Subsequently, we integrated these targets within the *pancreatic cancer* pathway and the *complement and coagulation cascades* pathway from KEGG (Table 4). Noticeably, we identified an important number of validated targets of these miRNAs in both pathways clearly related to PDAC, DECC and VTE. Moreover, we further identified a group of predicted targets whose regulation by these miRNAs could be experimentally demonstrated in future studies.

Similarly, we identified the targets of the 7 miRNAs that were down-regulated in the VTE group in a sample right before the VTE event, and compared it with the sample obtained at inclusion (Table 5). Again, we identified a great number of validated and predicted targets of these down-regulated miRNAs that could shed light on the mechanisms triggering the VTE event in these patients.

### 2.5. Neutrophil Activation Markers and Risk of Thrombosis

Using the Wilcoxon-Mann-Whitney test, we analyzed the differences in the levels of each marker of neutrophil activation between patients who developed VTE and those who did not. The neutrophil counts at inclusion were similar in the two groups (Table 1). We observed an increase in calprotectin levels in those patients who developed VTE (1374 ng/mL) compared with those who did not (427 ng/mL) (*p* = 0.017) and also in plasma myeloperoxidase (MPO) levels (98 vs. 87 ng/mL, respectively; *p* = 0.059). No substantial differences were observed between groups in the plasma levels of nucleosomes (0.12 vs. 0.096 U, respectively; *p* = 0.25) or cfDNA (2048 vs. 1688 ng/mL, respectively; *p* = 0.41). Furthermore, to evaluate whether the different markers of neutrophil activation measured in plasma have the same cellular origin, we evaluated their correlation. A significant correlation was observed between calprotectin and MPO levels (Spearman r = 0.648, *p* < 0.001), between calprotectin and cfDNA levels (Spearman r = 0.594, *p* < 0.001), between MPO and cfDNA levels (Spearman r = 0.587, *p* = 0.001), between calprotectin and nucleosomes levels (Spearman r = 0.467, *p* = 0.009) and between cfDNA and nucleosomes levels (Spearman r = 0.359, *p* = 0.044).

**Figure 2:**
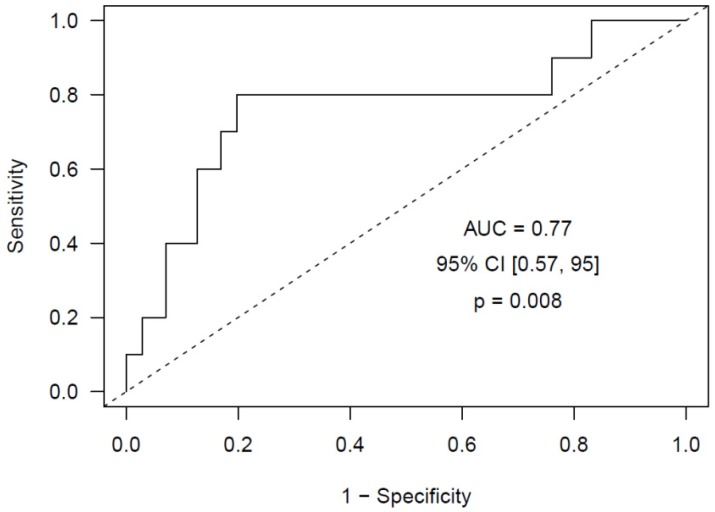
ROC curve obtained using the Elastic Net model that includes calprotectin as risk predictor of future VTE in PDAC and DECC patients at diagnosis.

To evaluate the ability of neutrophil activation markers to identify PDAC and DECC patients at high risk of developing VTE during follow-up, we conducted a Cox regression survival model with a time-dependent covariate, including the markers of neutrophil activation measured in each sample collected from the patients in rank form. We observed that, for each unit that the logarithm of the calprotectin concentration increases, the VTE risk in PDAC and DECC patients increases 6 times (*p* = 0.009). Next, we adjusted an Elastic Net logistic regression model and obtained a predictive model of VTE with calprotectin as predictor (AUC = 0.77, 95% CI (0.57, 0.95), *p* = 0.008), optimism-corrected AUC = 0.76 (Figure 2). The other neutrophil activation markers showed no significant predictive capacity of VTE.

## 3. Discussion

Pancreatic cancer induces a hypercoagulable state mainly mediated by a high tumoral expression of tissue factor, the activation of leukocytes with the release of NETs, the dissemination of tumor-derived microvesicles that promote hypercoagulability and an increased platelet activation [3]. In fact, pancreatic cancer bears the highest incidence of VTE complications [4], while cholangiocarcinoma is almost the same [9]. The appearance of disabling co-morbidities and a potential increase in future vascular thromboembolic events [1,5,34] brings about a reduction in the overall survival. Several scores have been proposed to evaluate the thrombotic risk of cancer patients [10,11,12,13,14,15], like the widely used Khorana score [10]. However, several limitations have been raised [12,16,17,18,19,20], thus creating the need to develop novel tools to predict and monitor the thrombotic risk [21].

Numerous studies conducted over the past decade have revealed that aberrantly expressed miRNAs are a hallmark of cancer and many other diseases. Thereby, the expression profile of miRNAs has been associated with tumor development, progression and response to therapy, suggesting their possible use as diagnostic, prognostic and predictive biomarkers [35]. Moreover, previous evidence shows that miRNAs can function as potential oncogenes or oncosuppressor genes by targeting each of the essential features described of cancer progression: Self-sufficiency in growth signals, insensitivity to anti-growth signals, apoptosis evasion, limitless replicative potential, sustained angiogenesis and tissue invasion and metastasis [35]. Furthermore, cancer metastasis is facilitated by miRNAs encapsulated in extracellular vesicles, released by primary tumor cells in order to create a cancer microenvironment for their progression [36].

In the present study we have identified a miRNA profile at diagnosis able to predict the occurrence of a VTE event in PDAC and DECC patients during follow-up. This model includes 7 miRNAs (miR-486-5p, miR-106b-5p, let-7i-5p, let-7g-5p, miR-144-3p, miR-19a-3p and miR-103a-3p) and achieves a high predictive capacity (ROC curve AUC = 0.95, 95% CI (0.87, 1), *p* < 0.001). Furthermore, applying this predictive model, we estimated the thrombotic risk of each PDAC and DECC patient of our study at inclusion, and the median thrombotic risk of the group of patients who suffered a VTE during follow-up was 0.72, while it was only 0.13 in the group of patients who did not suffer a VTE during follow-up (*p* < 0.0001), thus confirming the predictive ability of our model. Our promising results support the accomplishment of validation studies in an independent cohort of prospectively recruited biliopancreatic cancer patients.

Additionally, we aimed to identify up- or down-regulated miRNAs that could be involved in triggering the VTE event in PDAC and DECC cancer patients. In that respect, we studied the expression profile of miRNAs in the sample right before the VTE event compared with that obtained at inclusion. We identified a profile of 7 down-regulated miRNAs (miR-30e-3p, let-7i-5p, let-7g-5p, miR-144-3p, miR-199a-3p, miR-101-3p and miR-15a-5p) that might prompt the VTE event in these patients during follow-up. Among these, miR-144-3p and let-7g-5p showed the greatest differences in expression levels between both samples studied, what could indicate a greater involvement of these two miRNAs in triggering the VTE event in PDAC and DECC patients.

In order to further understand the biological mechanism potentially dysregulated by these miRNAs, we identified their targets and those pathways where they participate. Remarkably, most miRNAs were comprised in the predictive model or were down-regulated in the VTE patients, before the event, have targets involved in the *pancreatic cancer* pathway and in the *complement and coagulation cascades* that have been validated in previous studies. This fact reinforces the potential regulatory role of these miRNAs in PDAC and DECC patients. Henceforth, we will discuss several of these regulations in detail.

*KRAS*, *TP53* and *SMAD* act as oncogenes in pancreatic cancer. In fact, pancreatic adenocarcinoma is characterized by several germline or acquired genetic mutations, the most common being *KRAS* (90%), *CDK2NA* (90%), *TP53* (75–90%), *SMAD4*/*DPC4* (50%). Thus, the diagnostic and prognostic value of the mutational status is currently under evaluation [37] and may represent a future therapeutic target [38]. Furthermore, this tumor type holds epigenetic alterations that could guide personalized cancer therapies. In addition, the tumor microenvironment, the chemo-resistant cancer stem cells, and the desmoplastic stroma have been the target of recent clinical investigations [39,40,41,42]. Two of the miRNAs included in our VTE predictive model and down-regulated in VTE patients, let-7g-5p and let-7i-5p, belong to the let-7 miRNA family that regulates *RAS*. Gain-of-function approaches have shown that miRNAs of the let-7 miRNA family act as tumor suppressors by targeting oncoproteins with crucial roles in various cancer pathways, such as *RAS* [35]. Moreover, the combination of let-7i, miR-142, miR-26a and miR-141 has been proposed as prognostic model to robustly stratify nasopharyngeal carcinoma patients into high- and low- risk groups of distant metastasis [43].

A common characteristic of many cancer cells is the mutational status of the tumor suppressor gene *TP53*, with almost half of human malignancies harboring an altered form of this gene [44]. Four miRNAs comprised in our VTE predictive model (miR-106b-5p, let-7i-5p, let-7g-5p and miR-19a-3p) have *TP53* as validated or predicted target. 

As observed, the regulation of the human biological pathways is very complex since one miRNA usually targets many mRNAs in the same pathway and every mRNA is targeted by many miRNAs to ensure a fine-tuned global regulation. Furthermore, the targets regulated by each miRNA may have opposite functions, which represents a controversy on whether the final outcome of a miRNA would then be oncogenic or tumor suppressive. It is now known that the miRNA may produce an overall net oncogenic or net tumor suppressive effect, depending on the balance between miRNA-mediated upregulation or downregulation of oncogenic and tumor suppressive pathways, as well as the effects of the miRNA on cancer-immune system interactions and various other tumor-modifying extrinsic factors [45].

Additionally, in vitro studies have demonstrated that several miRNAs comprised in our VTE predictive model and those down-regulated miRNAs that could be involved in triggering the VTE event in PDAC and DECC patients, target proteins involved in coagulation, such as serpins (plasminogen activator inhibitor-1, miR-486-5p; urokinase, miR-19a-3p) and coagulation factors (tissue factor, miR-106b-5p; fibrinogen alpha and beta and gamma, miR-144-3p) [46,47,48,49,50]. Noticeably, other predicted targets involved in coagulation may be regulated by these miRNAs, such as serpins, thrombomodulin, von Willebrand factor, other coagulation factors, etc. Future studies conducted in vitro in cell cultures and in vivo in animal models would verify the regulation of these predicted targets and would elucidate the degree of participation of each miRNA in the final complex regulatory mechanisms exerted by these miRNAs in PDAC and DECC patients. To the best of our knowledge, this is the first study in which the predictive role of miRNAs for VTE in PDAC and DECC patients is addressed.

Upon activation, neutrophils release their content through different mechanisms like degranulation and NETosis, thus prompting thrombosis. Hence, in our study we also explored the ability of several markers of neutrophil activation, in order to identify PDAC and DECC patients at high risk of developing VTE during follow-up. To that end, we measured different plasma markers of neutrophil activation following the strategy addressed in previous studies [51,52,53,54,55,56,57]. We have observed an increase in calprotectin and MPO plasma levels in those patients who developed VTE compared with those who did not. Nucleosomes and cfDNA levels were also slightly increased in these patients. The neutrophil activation markers studied, herein, could have a different cellular origin other than neutrophils. Calprotectin and MPO could be released by monocytes, macrophages or eosinophils, but only to a lesser extent. In fact, calprotectin accounts for approximately 60% of total soluble proteins in the cytosolic fraction of neutrophils [58] and, although, low levels are found in other phagocytic cells, it is clinically considered to be neutrophil-specific and higher levels in plasma or feces are found in diseases associated with increased neutrophil activity. cfDNA and nucleosomes could be released into plasma by apoptotic or necrotic cells which, not least, may be present in these cancer patients. However, all these four markers were significantly correlated pairwise, indicating that they all had, to some extent, the same origin, probably an increased activation of neutrophils or NETs formation. Although, only calprotectin and MPO were significantly increased. It could be speculated that the level of neutrophils may affect the levels of plasma activation markers, but no differences in neutrophil counts were observed between VTE and non-VTE patients (Table 1). Several patients were treated with gemcitabine, a cytostatic agent that may lower the neutrophil count and, by that, interfere with the extent of neutrophil activation markers. However, no correlation was observed between changes in neutrophil counts during treatment and the level of neutrophil activation markers, thus discarding this effect in our study. Furthermore, a greater proportion of VTE patients (7 of 10) than non-VTE patients (11 of 22) received this treatment, so it would tend to reduce the difference in plasma markers between the two groups.

Regarding the predictive ability of these neutrophil activation markers, we observed that for each unit that the logarithm of the calprotectin concentration increased, the VTE risk in PDAC and DECC patients increased six times. Finally, we obtained a predictive model for VTE with calprotectin as a predictor, which achieved an AUC = 0.77 (95% CI (0.57, 0.95), *p* = 0.008). To date, only one study has explored the ability of markers of NETs to predict a VTE event in pancreatic cancer patients [57], where an increase in these markers was associated with the occurrence of VTE. Additionally, Jin et al. [59] observed that NETs markers predicted poor postsurgical survival of patients with PDAC. Furthermore, the incorporation of these markers with the standard TNM stating system refined the risk stratification and predicted survival in PDAC with improved accuracy.

A limitation of our study is the rather small sample size studied. However, the thorough clinical assessment of cancer patients for VTE every three months during two years hinders the recruitment and management of a high number of patients for this type of prospective studies. Nevertheless, consistent with the frequency of VTE events in biliopancreatic cancer patients, only 10 patients developed a VTE during follow-up. Thus, following common practice [60], a ratio of 2 controls per 1 patient was established in our case-control study. The validation of our results in an independent external cohort of biliopancreatic cancer patients prospectively recruited and followed would definitively reinforce our findings. The strengths of our study is that we conducted a thorough evaluation of patients at inclusion and during follow-up.

In conclusion, our study has highlighted the ability of plasma miRNAs and calprotectin as biomarkers for predicting a VTE event in PDAC and DECC patients. We have obtained and confirmed a profile of 7 miRNAs (miR-486-5p, miR-106b-5p, let-7i-5p, let-7g-5p, miR-144-3p, miR-19a-3p and miR-103a-3p) able to estimate the risk of future VTE in PDAC and DECC patients at diagnosis. These miRNAs are deeply involved in the *pancreatic cancer* pathway and the *complement and coagulation and cascades* pathways. Similarly, plasma calprotectin may be a valuable tool to estimate the VTE risk in these patients. Personalized medicine and targeted therapy have presently become the cornerstone in medicine. Thus, once validated in a larger independent cohort of patients, our predictive models may be implemented into daily clinical practice. The estimation of the thrombotic risk of each PDAC and DECC patient at diagnosis might promote a closer follow-up and a personalized thromboprophylaxis in high-risk patients.

Moreover, we have observed that 7 miRNAs (miR-30e-3p, let-7i-5p, let-7g-5p, miR-144-3p, miR-199a-3p, miR-101-3p and miR-15a-5p) are significantly down-regulated in PDAC and DECC patients right before the VTE event compared with inclusion at diagnosis. These miRNAs have highlighted the aforementioned target proteins and the mechanism that might be triggering a VTE in PDAC and DECC patients. Interestingly, four of these seven miRNAs are dysregulated in both analyses (miR-30e-3p, let-7i-5p, let-7g-5p and miR-144-3p), upregulated in VTE patients at inclusion compared with non-VTE patients and down-regulated right before the VTE event compared with inclusion. Particularly, miR-144-3p and let-7g-5p showed the greatest differences in the expression level right before the VTE event, what reinforces the idea of these miRNAs being strong candidates for prompting a thrombotic complication in PDAC and DECC patients. Future studies in different thrombotic scenarios would shed light in shared dysregulated mechanisms between pathologies of different etiology but with a common outcome.

## 4. Materials and Methods

### 4.1. Study Subjects

A total of 121 patients admitted on suspicion of an upper gastrointestinal cancer were prospectively recruited and followed for two years, between February 2008 and February 2011, at the Department of Gastrointestinal Surgery of the Aalborg University Hospital (Aalborg, Denmark) [33]. Cancer was confirmed histologically and staged according to the UICC6 system by means of diagnostic computer tomography (CT) of the thorax and abdomen, or positron emission tomography-CT (PET-CT). During follow-up, patients were objectively assessed for DVT by bilateral compression ultrasound and PE by an arterial-stage scan covering the pulmonary arteries (a CT pulmonary angiogram) as previously described [33] every three months, beginning at the time of cancer diagnosis. Patients who underwent curatively intended surgery were examined preoperatively and postoperatively.

The exclusion criteria were: Previous (within the past three years) or concomitant cancer of any origin; known immunologic connective tissue disease; mental disorder; previous episodes of VTE; and treatment with heparin, low molecular weight heparin (LMWH) or vitamin K antagonists at the time of inclusion in the study. Patients who did not provide consent because of debilitation, advanced age or refusal to participate in the study were also excluded. All patients provided written and oral informed consent (Clinical Trials.gov: NCT00660205; approval of local ethics committee of Region North Jutland, Denmark: N-20080002). The study was performed according to the declaration of Helsinki, as amended in Edinburgh in 2000.

### 4.2. Blood Collection

Blood was drawn from all patients at diagnosis and every three months during follow-up. Blood was collected in Monovette tubes (Sarstedt, North Rhine-Westphalia, Germany) containing 0.109 M trisodium citrate and then centrifuged at 2600× *g* for 20 min at 4 °C. Plasma was collected ensuring the absence of platelet and leukocyte contamination, by only taking the 2/3 of upper plasma, and stored in aliquots at −80 °C until used.

### 4.3. RNA Isolation

Total plasma RNA (including miRNAs) was isolated using the miRNeasy Mini Kit (Qiagen, Hiden, Germany), following manufacturer’s instructions with several modifications optimized by our group [61]. During the isolation, an RNA carrier (tRNA, Ambion, Bleiswijk, The Netherlands) was included to enhance the yield, and a mixture of synthetic miRNAs (Spike-in kit UniRT, Exiqon, Vedbaek, Denmark) was included to control for RNA isolation efficiency and cDNA synthesis. The concentration and purity of the RNA was assessed by spectrophotometric quantification with the NanoDrop ND-1000 (Thermo Fisher Scientific, Wilmington, DE, USA). RNA was stored at −80 °C until used. The haemolysis of plasma was assessed by measuring the absorbance of the haemoglobin at 412 nm.

### 4.4. Quantification of the Expression Level of miRNAs

The expression level of miRNAs was quantified by real-time quantitative reverse transcription PCR (RT-qPCR) in two stages:

#### 4.4.1. Screening Stage

Based on the quality of the isolated RNA, 5 patients who suffered a VTE event during follow-up (VTE group) and five who did not were selected and miRNAs expression level was studied at inclusion. Additionally, in the VTE patients, the expression level of miRNAs was studied in the last plasma sample obtained right before the VTE event to identify dysregulated miRNAs that may prompt the VTE event. The Universal cDNA Synthesis Kit II (Exiqon, Vedbaek, Denmark) was used for the retrotranscription and the expression level of 179 candidate miRNAs mostly present in plasma was quantified with the Serum/Plasma Focus microRNA PCR Panel V4 (Exiqon, Vedbaek, Denmark) and the ExiLENT SYBR Green Master Mix (Exiqon, Vedbaek, Denmark) in a LightCycler 480 II (Roche, Mannheim, Germany). Furthermore, each panel includes the following internal controls: five synthetic RNAs of the RNA Spike-in-kit aimed to monitor the RNA isolation and cDNA synthesis, and an inter-plate calibrator in triplicate and a negative control to evaluate qPCR performance.

To normalize the expression level of each miRNA, the best endogenous reference with the highest stability and the lowest biological variance among all samples was selected. The candidate normalization miRNAs proposed by the Serum/plasma Focus microRNA PCR Panel V4 were miR-423-5p, miR-425-5p, miR-93-5p, miR-191-5p and miR-103a-3p. To select the best reference, the comprehensive tool RefFinder was employed which integrates the computational programs geNorm, Normfinder, BestKeeper and the comparative delta-Ct method (https://www.heartcure.com.au/for-researchers/). The miRNA expression levels were normalized by the 2^−ΔΔCT^ method.

Next, a multivariable logistic regression model was generated, able to accurately predict a VTE event during follow-up using the data of both patient groups at inclusion. Additionally, those miRNAs dysregulated in a sample right before the VTE event, compared with the sample obtained at inclusion in the VTE group were identified.

#### 4.4.2. Confirmation Stage

Once selected a miRNA profile potentially able to predict a VTE event in PDAC and DECC patients during follow-up, their expression level was quantified in a larger cohort of patients (26 patients with PDAC and 6 with DECC, 10 who developed a VTE during follow-up [9 PDAC and 1 DECC], and 22 age- and sex-matched who did not) at inclusion by RT-qPCR in duplicate. For that aim, specific primers for each miRNA, miRCURY LNA miRNA PCR Assay (Exiqon, Vedbaek, Denmark) were used. Each measurement in duplicate was considered suitable when the standard deviation (SD) was <0.5.

### 4.5. Identification of the miRNAs’ Targets

Once the dysregulated miRNAs were detected, the identification of their predicted and validated target proteins related to cancer and VTE was conducted by using the databases miRWalk 2.0 (http://zmf.umm.uni-heidelberg.de/apps/zmf/mirwalk2/) and Kyoto Encyclopedia of Genes and Genomes (KEGG, Kyoto, Japan) (https://www.genome.jp/kegg/). miRWalk 2.0 (Mannheim, Germany) combines information from 12 existing miRNA-target prediction programs (DIANA-microTv4.0, DIANA-microT-CDS, miRanda-rel2010, mirBridge, miRDB4.0, miRmap, miRNAMap, doRiNA i.e.,PicTar2, PITA, RNA22v2, RNAhybrid2.1 and Targetscan6.2). Finally, the targets obtained with miRWalk 2.0 were integrated within the *pancreatic cancer* pathway and the *complement and coagulation cascades* pathway from KEGG.

### 4.6. Quantification of Neutrophil Activation Markers

Different markers of neutrophil activation were measured in every plasma sample obtained during follow-up from the 32 patients studied herein, following the strategy addressed in previous studies [51,52,53,54,55,56,57]. Cell-free DNA (cfDNA; Quant-iT PicoGreen dsDNA kit, Life Technologies, Eugene, OR, USA) and nucleosomes (Cell Death Detection ELISA^PLUS^ kit, Roche, Mannheim, Germany) were measured as markers of the nuclear content of neutrophils released by neutrophils upon NETosis. Calprotectin (Human Calprotectin ELISA kit, Hycult Biotech, Uden, The Netherlands) was measured as a marker of cytoplasmic content and MPO (Human MPO ELISA kit, Abnova, Taoyuan, Taiwan) as a marker of the content of neutrophil granules, were both released upon neutrophil activation by different mechanisms. In all cases the experiments were performed following the manufacturer’s instructions.

### 4.7. Statistical Analysis

All statistical analyses were performed using R (version v3.5.1; Vienna, Austria). Continuous variables were presented as median and interquartile range, and categorical variables as count and percentage. In the screening stage, an elastic net logistic regression model for VTE risk was adjusted using the miRNA expression levels at inclusion of 10 selected patients (five who developed VTE and five without VTE). The predictive ability of the model was assessed by estimating an optimism-corrected area under the curve (AUC) for the receiver operator characteristic (ROC) analysis, and using 1000 bootstrap replicates in the screening stage. Next, this AUC was verified in the confirmation stage. The formula to calculate the risk of VTE in each patient was built with the coefficients rendered by the model for each predictive variable. Furthermore, a paired T-test was applied to identify dysregulated miRNAs in the sample right before the VTE event compared with that obtained at inclusion. The association of neutrophil activation markers with VTE was assessed by comparing the levels of each marker in both clinical groups (with and without VTE) by using the Wilcoxon-Mann-Whitney test. The ability of neutrophil activation markers to identify PDAC and DECC patients, at high risk of developing VTE during follow-up, was evaluated by means of a Cox regression survival model with a time-dependent covariate, including the neutrophil activation markers measured in each sample of the patients in rank form. The results were considered statistically significant at *p* < 0.05. Additionally, an elastic net logistic regression model for VTE risk was adjusted using the levels of neutrophil activation markers in every sample obtained from the 32 patients at inclusion and during follow-up. For this case-control study, a ratio of two controls per one case was established. Accordingly, the 10 patients who developed VTE during follow-up were selected, along with a selection of 22 patients who did not develop VTE, age- and sex-matched (two extra controls to make the control group more comparable to the VTE-group) and representative of the whole sample set.

## Figures and Tables

**Table 1:** Characteristics of the 32 patients with pancreatic ductal adenocarcinoma (PDAC) and distal extrahepatic cholangiocarcinoma (DECC) patients studied.

	VTE Patients	Non-VTE Patients	Statistical Significance *p*
**N (% of total)**	10 (31.3)	22 (68.8)	-
**Age, y, median (range)**	64 (50–79)	66 (51–84)	0.57
**Female sex, N (%)**	4 (40)	9 (40.4)	0.64 *
**Time to VTE, months, median (range)**	3 (1–24)		
**Tumor location**			
**PDAC N (%)**	9 (90)	17 (77.3)	
**DECC N (%)**	1 (10)	5 (22.7)	0.64 *
**Leukocyte count**			
**Neutrophil count**	8.6 ± 3.2 × 10^9^/L	8.7 ± 2.5 × 10^9^/L	0.92
**(Mean ± SD)**	6.6 ± 3.6 × 10^9^/L	6.1 ± 2.5 ×10^9^/L	0.65
**Treatment**			
**Curative intended surgery**	2	11	
**Neoadjuvant treatment**	1	0	
**Postop. Chemotherapy**	0	3	
**Palliative gemcitabine**	7	11	0.12 *
**UICC stage**			
**I**	1	4	
**II**	2	6	
**III**	2	5	
**IV**	5	7	0.83 *
**WHO performance score**			
**0**	3 (30)	18 (81.8)	
**1**	5 (50)	3 (13.6)	
**2**	2 (20)	1 (4.6)	0.012 *
**CCI score**			
**0**	5 (50)	18 (81.8)	
**1**	2 (20)	3 (13.6)	
**2**	2 (20)	1 (4.6)	
**4**	1 (10)	0	0.14 *
**Khorana score**			
**2**	3 (30)	9 (40.9)	
**3**	4 (40)	8 (36.4)	
**4**	3 (30)	4 (18.2)	
**5**	0	1 (4.6)	0.92 *

UICC, Union for International Cancer Control, 6th Edition; WHO, World Health Organization; CCI score, Charlson Comorbidity Index score. Mann-Whitney. * Fisher’s exact test.

**Table 2:** miRNAs comprised in the predictive model of VTE in PDAC and DECC patients obtained in the screening stage. miRNA sequences according to miRBase 22.1. Fold-change is defined as the ratio of the average expression level of a miR in PDAC and DECC patients who suffered a VTE event during follow-up and those who did not.

miRNA	Sequence	Fold-Change	Coefficient
hsa-miR-486-5p	uccuguacugagcugccccgag	1.82	0.041
hsa-miR-32-5p	uauugcacauuacuaaguugca	2.60	0.082
hsa-miR-106b-5p	uaaagugcugacagugcagau	1.96	1.235
hsa-miR-326	ccucugggcccuuccuccag	−2.58	−0.761
hsa-let-7i-5p	ugagguaguaguuugugcuguu	1.87	0.668
hsa-let-7g-5p	ugagguaguaguuuguacaguu	1.74	0.066
hsa-miR-144-5p	ggauaucaucauauacuguaag	3.57	2.509
hsa-miR-144-3p	uacaguauagaugauguacu	4.28	0.166
hsa-miR-19a-3p	ugugcaaaucuaugcaaaacuga	1.51	0.201
hsa-miR-103a-3p	agcagcauuguacagggcuauga	1.73	0.284
hsa-miR-30e-3p	cuuucagucggauguuuacagc	2.63	1.820

**Table 3:** Dysregulated miRNAs in PDAC and DECC patients who develop a VTE during follow-up, comparing the sample at inclusion and that right before the VTE event. miRNA sequences according to miRBase 22.1. Delta is defined as the difference of the average expression level of a miRNA between the samples at inclusion and the ones right before the VTE event. The negative values of delta represent down-regulation of miRNAs in the samples right before the VTE event.

miRNA	Sequence	*p* (*t*-Test)	Delta
hsa-miR-30e-3p	cuuucagucggauguuuacagc	0.015	−0.035
hsa-let-7i-5p	ugagguaguaguuugugcuguu	0.026	−0.062
hsa-let-7g-5p	ugagguaguaguuuguacaguu	0.03	−0.34
hsa-miR-144-3p	uacaguauagaugauguacu	0.03	−0.8
hsa-miR-199a-3p	acaguagucugcacauugguua	0.025	−0.11
hsa-miR-101-3p	uacaguacugugauaacugaa	0.029	−0.26
hsa-miR-15a-5p	uagcagcacauaaugguuugug	0.031	−0.07

**Table 4:** Validated and predicted targets of the 11 miRNAs included in the predictive model of VTE in PDAC and DECC patients at inclusion. These target proteins were identified using miRWalk 2.0 and were further integrated within the *pancreatic cancer* pathway and the *complement and coagulation cascades* pathway from KEGG. Validated targets are those that have been empirically validated to be regulated by a miRNA. Predicted targets are those that have been theoretically estimated based on the free binding energy between a miRNA and a putative target mRNA sequence.

	*Pancreatic Cancer* Pathway		*Complement and Coagulation Cascades* Pathways	
miRNA	Validated Target	Predicted Target	Validated Target	Predicted Target
**hsa-miR-486-5p**	-	CDK4	SERPINE1	F2R, F9, C6, C8A, PLAT, C5AR1, SERPING1
**hsa-miR-32-5p**	-	MAPK8, PIK3CB, BRAF, CASP9, PLD1, CDC42	-	-
**hsa-miR-106b-5p**	ACVR1B, CCND1, CDC42, E2F1, E2F2, E2F3, JAK1, MAPK1, MAPK9, RB1, SMAD4, STAT3, TGFBR2, TP53, VEGFA	BRAF, KRAS	F2R, F3	CD46, C5
**hsa-miR-326**	AKT1, CCND1, ERBB2, KRAS	TGFA, PGF, CDKN2A, RAC2, MAPK10	C1R, F9	BDKRB2, C8G, C2, SERPINF2, C8B, C1S, MASP1
**hsa-let-7i-5p**	CCND1	MAPK8, AKT2, BCL2L1, TP53	CD59	-
**hsa-let-7g-5p**	AKT2, BCL2L1, CCND1, CDKN2A, KRAS, SMAD2, TGFBR1	MAPK8, TP53	CD59	-
**hsa-miR-144-5p**	-	STAT1, STAT3, E2F3	-	F2R
**hsa-miR-144-3p**	RAC1, TGFB1	STAT1, E2F3, MAPK9, CDC42, AKT2, PIK3CG	FGA, FGB, FGG	F13B, PLAT, PLG, CR1, CR2
**hsa-miR-19a-3p**	AKT1, CCND1, MAPK1, PIK3R3, RAF1, SMAD4, TGFBR2, TP53	CCND1, RAF1, PIK3CA, PIK3R1	PLAU	TFPI, CR2, C7, F3, PLAU, THBD, C6, CD55, SERPIND1, BDKRB2
**hsa-miR-103a-3p**	CDK6, PIK3R1, RAD51	SMAD4, PLD1, FIGF, RALBP1, CDC42, MAPK3, IKBKG, RALGDS	-	C1QB, MASP1, SERPING1, VWF, C1S, SERPINC1, CR2
**hsa-miR-30e-3p**	KRAS	MAPK10, RALBP1, ERBB2, RALB, CASP9, RAD51	C6	C1S, FGG

**Table 5:** Validated and predicted target genes of the 7 miRNAs down-regulated in the VTE group of patients in a sample right before the VTE event compared with the sample obtained at inclusion. These target proteins were identified using miRWalk 2.0 and were further integrated within the *pancreatic cancer* pathway and the *complement and coagulation cascades* pathway from KEGG. Validated targets are those that have been empirically validated to be regulated by a miRNA. Predicted targets are those that have been theoretically estimated based the free binding energy between a miRNA and a putative target mRNA sequence.

	*Pancreatic Cancer* Pathway		*Complement and Coagulation Cascades* Pathways	
miRNA	Validated Target	Predicted Target	Validated Target	Predicted Target
**hsa-miR-30e-3p**	KRAS	MAPK10, RALBP1, ERBB2, RALB, CASP9, RAD51	C6	C1S, FGG
**hsa-let-7i-5p**	CCND1	MAPK8, AKT2, BCL2L1, TP53	CD59	-
**hsa-let-7g-5p**	AKT2, BCL2L1, CCND1, CDKN2A, KRAS, SMAD2, TGFBR1	MAPK8, TP53	CD59	-
**hsa-miR-144-3p**	RAC1, TGFB1	STAT1, E2F3, MAPK9, CDC42, AKT2, PIK3CG	FGA, FGB, FGG	F13B, PLAT, PLG, CR1, CR2
**hsa-miR-199a-3p**	AKT1, E2F2, MAPK1, MAPK8, MAPK9	CDC42	-	C4BPA, PLG, C3AR1
**hsa-miR-101-3p**	E2F3, MAP2K1, RAC1, TGFBR1, TGFBR2, VEGFA	ACVR1C, BRAF, EGFR, PLD1, CDC42, AKT2, PIK3CG	CD46	FGA, CR2, F13B, PLAT, PLG
**hsa-miR-15a-5p**	ACVR1B, AKT3, CCND1, CDK6, CHUK, E2F3, IKBKG, NFKB1, PIK3R1, SMAD3, TP53, VEGFA	SMAD4, IKBKB, MAP2K1, RAF1, ARHGEF6	-	-
